# Supplementary material for: Seroprevalence of SARS-CoV-2 IgG Antibodies and Factors Associated with SARS-CoV-2 IgG Neutralizing Activity among Primary Health Care Workers 6 Months after Vaccination Rollout in France
Source: Viruses. 2022 May 3;14(5):957. doi: 10.3390/v14050957 (PMC9148144; doi:10.3390/v14050957)
Supplement: Supplementary file 1 [file viruses-14-00957-s001.zip › viruses-1687215-supplementary.pdf]

Supplementary Material

Table S1. Name of the vaccine received by the PHCWs (N = 1612; COVID-SéroPRIM study, France, 2021).

**Vaccine name**	***n***	**%**
One dose (N = 186)		
BNT162b2	122	65.6
AZD1222	38	20.4
mRNA-1273	22	11.8
Ad26.COV2.S	4	2.1
Two doses (N = 1277)		
BNT162b2 × 2	901	70.6
AZD1222 × BNT162b2	262	20.5
Others	114	8.9
Three doses (N = 2)		
BNT162b2 × 3	1	50.0
AZD1222 × BNT162b2 × mRNA-1273	1	50.0
NA	15	
